# The impact of a simulated intervention on attitudes of undergraduate nursing and medical students towards end of life care provision

**DOI:** 10.1186/s12904-016-0143-2

**Published:** 2016-08-02

**Authors:** Claire Lewis, Joanne Reid, Zara McLernon, Rory Ingham, Marian Traynor

**Affiliations:** 1 Northern Ireland Biobank, Queen’s University Belfast, Belfast, UK; 2 School of Nursing and Midwifery, Queen’s University Belfast, Belfast, UK; 3 School of Medicine, Dentistry and Biomedical Sciences, Belfast, UK

**Keywords:** Nursing students, Medical students, Undergraduate, Simulation, End of life, Intervention, Attitudes

## Abstract

**Background:**

The concerns of undergraduate nursing and medical students’ regarding end of life care are well documented. Many report feelings of emotional distress, anxiety and a lack of preparation to provide care to patients at end of life and their families. Evidence suggests that increased exposure to patients who are dying and their families can improve attitudes toward end of life care. In the absence of such clinical exposure, simulation provides experiential learning with outcomes comparable to that of clinical practice. The aim of this study was therefore to assess the impact of a simulated intervention on the attitudes of undergraduate nursing and medical students towards end of life care.

**Methods:**

A pilot quasi-experimental, pretest-posttest design. Attitudes towards end of life care were measured using the Frommelt Attitudes Towards Care of the Dying Part B Scale which was administered pre and post a simulated clinical scenario. 19 undergraduate nursing and medical students were recruited from one large Higher Education Institution in the United Kingdom.

**Results:**

The results of this pilot study confirm that a simulated end of life care intervention has a positive impact on the attitudes of undergraduate nursing and medical students towards end of life care (*p* < 0.001).

**Conclusions:**

Active, experiential learning in the form of simulation teaching helps improve attitudes of undergraduate nursing and medical students towards end of life. In the absence of clinical exposure, simulation is a viable alternative to help prepare students for their professional role regarding end of life care.

## Background

End of life care is a known area of concern for undergraduate nursing and medical students with reports of anxiety [1], dread [2], and emotional distress [3] when faced with the experience of providing care to a dying patient and their family. Amongst nursing students commonly voiced concerns include knowing how to communicate effectively with patients and their families; their own emotional reaction to death and practical issues such as symptom management and care of the body after death [1, 4]. Similarly, medical students have reported feeling ‘out of their depth’ when caring for dying patients and their families [5] and have voiced concern over the tension between their own professional detachment and emotional regard for patients and their families [6]. It is claimed that staff who have anxiety about death and dying are less comfortable providing end of life care [7] which may result in them distancing themselves from dying patients and their families in order to cope with their own fears and emotions. Attitudes towards death and dying can therefore directly impact on the quality of care a person receives at the end of life.

It has been suggested that both registered nurses and nursing students with greater experience of caring for patients who are dying have more favourable attitudes towards end of life care [8, 9] Similarly, it has been suggested that increased exposure to patients receiving end of life care will improve newly qualified doctors’ competence and confidence in delivering care to this patient group [10]. Yet many students may have limited opportunities in the course of their clinical placements to experience end of life care provision; or may be denied access by their peers who act as gatekeepers to the most unwell patients [5].

In the absence of such clinical practice experience, educators have been increasingly utilising simulation with high fidelity mannequins to provide experiential learning in end of life care education. This approach has been demonstrated to provide experiences and educational outcomes comparable to that of clinical placement [11]; allowing students to develop their practice skills and improve their critical thinking and decision making in a safe and non-threatening environment. From the limited evidence available, simulation has been proposed as a viable approach to end of life education given its positive impact on knowledge acquisition, communication skills, student satisfaction and level of engagement in learning [12]. To date however there has been a relative dearth of evidence on the impact of this approach on undergraduate nursing in the United Kingdom and substantially less on its impact when utilised as an interprofessional teaching strategy.

## Methods

### Objectives and design

The aim of this pilot study was to assess the impact of a simulated intervention on the attitudes of undergraduate nursing and medical students towards end of life care provision. The study used a quasi-experimental, pretest-posttest design without a control group.

### Setting, sample and recruitment

The study took place in one UK Higher Educational Institution (HEI) between March 2015 and May 2015 following ethical approval from the University’s School of Nursing and Midwifery Research Ethics Committee.

The sample population consisted of all third year nursing students and fourth year medical students enrolled in the University’s undergraduate nursing and medicine degree programmes. Recruitment commenced with the Chief Investigator (CI) providing a brief overview of the study to eligible students at the beginning of a large group lecture. This was followed up with an email (circulated by administrative staff on behalf of the CI) with an attached participant information sheet and contact details of the CI for those who wished to participate. Participation in the study was entirely voluntary and written informed consent was obtained from each student prior to the study commencing.

### Data collection and analysis

To measure student attitudes towards end of life care, participants were asked to complete the Frommelt Attitudes Towards Care of the Dying (FATCOD) Part B scale before and after the simulated experience [13]. This is a validated tool designed to measure non-family caregiver’s attitudes towards providing care to people who are terminally ill and their families. A non-family caregiver is defined as a person who is giving care to a dying person, professional or non-professional, who is not a member of the patient’s family [13]. The FATCOD Part B has been used widely to measure undergraduate nursing and medical students’ attitudes towards end of life care [14–17].

The FATCOD Part B instrument has 30 Likert-type items which are scored on a five-point scale from 1 (Strongly Disagree) to 5 (Strongly Agree). The instrument consists of equal numbers of positively and negatively worded items therefore higher scores reflect more positive attitudes. Validity and reliability of the FATCOD Part B has been demonstrated previously [13] and written permission to use the scale was obtained.

Data was analysed using the SPSS statistical package. Descriptive statistics were used to summarise and describe the data, and the paired *t* test used to compare mean pre and post intervention FATCOD scores.

### Intervention

Students volunteered to participate in a simulation session, intended to reflect the challenges of providing end of life care to terminally ill patients and their families. Each session consisted of two independent simulation scenarios, designed to be incremental in their level of complexity. Students participated in one scenario and observed one scenario. The simulated sessions were delivered in the School’s simulation facilities using a high fidelity simulator and a role player (simulated patient) as the patient’s relative.

The simulated scenarios ran for approximately 25 min each. They were designed to offer the students an opportunity to practice their skills of symptom assessment and management; their skills in recognising and responding to the physiological changes which may occur at end of life; and to develop therapeutic communication skills with patients and their families. The scenarios captured the patient journey from receiving bad news right through to the point of death. Prior to commencing the simulation, students were given a pre-brief or handover of the patient’s history and presenting complaint (Table 1); along with a set of case notes which had been put together for each patient. Immediately following each scenario, students participated in a debriefing session which was facilitated by members of the research team. Video recordings of the simulated scenarios were used during the debriefing to assist in student feedback and reflection.

**Table 1:** Example of simulation pre-brief

Symptom assessment and recognising/responding to signs of dying	
Background Patient name: John Smyth Age: 58 Next of Kin: Mary Smyth (Wife). Has one daughter (Jayne, 32) who lives in Australia. Consultant: Dr Jones Diagnosis: Stage IV Non-Small Cell Lung Cancer with bone and liver metastases. Diagnosed March 2014. Previously treated with palliative radiotherapy and chemotherapy. John is a 58 year old gentleman with advanced lung cancer. He has been an inpatient in Medical Ward 2 for five days; you are the nurse responsible for his care. John was admitted initially for treatment of a chest infection and symptom management of severe back pain and right-sided abdominal pain. On admission he was commenced on IV antibiotics for his chest infection and was reviewed by the palliative care nurse specialist who increased the dose of his analgesics for pain control. Since admission John’s condition has progressively deteriorated and he is now in the active phase of dying. John and Mary have spoken at length with medical and nursing staff about his wishes for end of life. It has been agreed that John should remain in hospital for his end of life care as Mary could not cope on her own at home. Their daughter Jayne is on her way home from Australia and is hoping to arrive tomorrow. John is now actively dying. He has been commenced on a syringe driver with 30mg of morphine for pain relief and midazolam 10mg for breathlessness. His wife is anxious that his breathing has become very rattley, she is concerned it is troublesome for John. She also wants to know how long John might have left as she is anxious her daughter could miss saying goodbye to her father.	

## Results

A total of 19 students participated in this pilot study; 15 nursing students and 4 medical students. Higher FATCOD scores indicate a more positive attitude towards providing care for patients who are dying and their families. The baseline pre-intervention FATCOD scores ranged from 100 to 141, with post-intervention scores ranging from 113 to 145 (Table 2).

**Table 2:** FATCOD scores pre and post intervention

Questionnaire	Pre Score	Post Score
1	116	125
2	123	129
3	124	131
4	135	139
5	100	119
6	136	145
7	117	135
8	131	141
9	115	137
10	114	121
11	108	118
12	102	119
13	123	120
14	111	135
15	117	118
16	141	136
17	108	113
18	119	126
19	133	133
Range	100–141	113–145
Mean	119.6	128.4
Standard Deviation	11.6	9.3

As depicted in Table 2, the pre-intervention FACTOD mean for the group was 119.6 (±11.6 with a maximum score of 150). The post-intervention FATCOD mean for the group was 128.4 (±9.3 with a maximum score of 150). Using a paired *t* test, a significant difference was found in the group’s mean FATCOD score after the intervention (t(18) = 4,29; *p* < 0.001), indicating a significantly more positive attitude towards end of life care as a result of the end of life simulation (Table 3).

**Table 3:** FATCOD mean scores pre and post intervention

Mean FATCOD scores		Mean change in difference	df	t
Pre Intervention	Post Intervention			
119.6	128.4	8.8***	18	4.29

***significant at *p* < 0.001 using the paired-*t* test

Mean scores for individual FATCOD items are presented in Table 4. Twenty six items had an increase in their mean scores post the simulated intervention; three items had a decrease (item numbers 9, 19 and 21) and one remained the same (item number 6). As can be seen in Table 2, all but two students (participants 13 & 16) had an increase in their FATCOD scores after the simulated intervention. Participant 16 had the highest pre-score of the group (141) indicating an already positive attitude towards end of life care prior to the simulation.

**Table 4:** FATCOD item data

FATCOD Item	Pre-Simulation		Post-Simulation		Difference in means
	Mean Score	Standard Deviation	Mean Score	Standard Deviation	
1. Giving care to the dying person is a worthwhile experience	4.68	0.75	4.89	0.32	0.21
2. Death is not the worst thing that can happen to a person	3.63	1.38	3.79	1.13	0.16
3. I would be uncomfortable talking about impending death with a dying person	2.74	1.15	3.58	1.02	0.84
4. Caring for the patient’s family should continue throughout the period of grief and bereavement	4.63	0.50	4.89	0.32	0.26
5. I would not want to care for a dying person	4.53	0.51	4.63	0.50	0.1
6. The non-family caregiver should not be the one to talk about death with the dying person	3.89	0.99	3.89	1.10	0
7. The length of time required to give care to a dying person would frustrate me	4.32	0.89	4.79	0.42	0.47
8. I would be upset when the dying person I was caring for, gave up hope of getting better	2.89	1.05	3.58	1.12	0.69
9. It is difficult to form a close relationship with the dying person	4.26	0.65	4.05	0.97	−0.21
10. There are times when death is welcomed by the dying person.	4.16	0.90	4.32	0.58	0.16
11. When a patient asks, “Am I dying?”, I think it is best to change the subject to something cheerful	4.16	0.83	4.53	0.61	0.37
12. The family should be involved in the physical care of the dying person if they want to	4.32	0.75	4.42	0.61	0.10
13. I would hope the person I’m caring for dies when I am not present	3.68	1.11	4.26	0.73	0.58
14. I am afraid to become friends with a dying person	3.84	1.12	4.21	0.63	0.37
15. I would feel like running away when the person actually died	4.00	0.94	4.47	0.70	0.47
16. Families need emotional support to accept the behaviour changes of the dying person	4.37	0.68	4.68	0.48	0.31
17. As a patient nears death, the non-family care-giver should withdraw from his/her involvement with the patient	3.89	0.94	4.00	0.88	0.11
18. Families should be concerned about helping their dying member make the best of his/her remaining life	3.79	0.79	3.89	1.05	0.10
19. The dying person should not be allowed to make decisions about his/her physical care.	4.68	0.75	4.53	0.61	−0.15
20. Families should maintain as normal an environment as possible for their dying member	3.79	0.79	4.16	0.83	0.37
21. It is beneficial for the dying person to verbalize his/her feelings.	4.37	0.60	4.16	0.83	−0.21
22. Care should extend to the family of the dying person	4.21	0.54	4.68	0.48	0.47
23. Care-givers should permit dying persons to have flexible visiting schedules	4.58	0.61	4.89	0.32	0.31
24. The dying person and his/her family should be the in-charge decision makers	3.68	0.95	3.84	0.96	0.16
25. Addiction to pain relieving medication should not be a concern when dealing with a dying person.	3.58	1.17	4.05	1.13	0.47
26. I would be uncomfortable if I entered the room of a terminally ill person and found him/her crying	3.37	1.12	3.95	0.85	0.58
27. Dying persons should be given honest answers about their condition	4.26	0.45	4.47	0.51	0.21
28. Educating families about death and dying is not a non-family care-givers responsibility	3.79	1.03	4.26	0.87	0.47
29. Family members who stay close to a dying person often interfere with the professionals’ job with the patient.	3.53	0.90	3.89	0.88	0.83
30. It is possible for non-family care-givers to help patients prepare for death	4.00	1.05	4.32	0.89	0.32

## Discussion

The results of this pilot study confirm that a simulated end of life care intervention has a positive impact on the attitudes of undergraduate nursing and medical students towards end of life care. As anticipated the simulated scenario provided an opportunity for the students to gain practical experience of providing end of life care through the use of a high fidelity simulator (patient at end of life) and role player (relative).

These results are consistent the growing body of literature which supports the use of end of life simulation in undergraduate nursing programmes. Quantitative [18], mixed methods [19], and qualitative studies [20, 21] have all reported positive outcomes for nursing students after exposure to an end of life simulated intervention. However there is currently less evidence to support its use with medical students or through IPE with other healthcare professionals. Bloomfield et al’s IPE study found simulation to be an effective means of preparing students to communicate with patients and relatives at end of life however their study focused solely on communication [22]. Bateman et al also investigated the use of simulation to evaluate communication between doctors and parents in paediatric end of life care [23]; however participants in their study were qualified medical staff rather than undergraduate medical students. In light of the results of this study, there is a clear need for greater emphasis on the use high fidelity simulators to teach end of life care in both undergraduate nursing and medical education and also to adopt an interprofessional approach. This is particularly relevant given that both medical students and nursing students who have experience of providing care to the dying report more positive attitudes towards end of life care [8, 10].

As presented in Table 3, a significant difference was found in the mean pre and post intervention FATCOD scores using the paired *t*-test. This finding is consistent with the results of a study by Lippe and Becker [14] who used simulation to deliver education about end of life care in the critical care setting to undergraduate nursing students only. They reported significantly improved mean FATCOD scores following their end of life simulation intervention. As outlined in Table 4, 26 out of 30 of the FATCOD items had an increase in their mean score post the end of life simulation. Notably, item 3 ‘I would be uncomfortable talking about impending death with a dying person’ had the greatest change in mean score. This is encouraging given that communication with a dying patient and their family is considered one of the top educational needs amongst nursing and medical staff [3, 24] Items with no change or a decrease in mean score post the intervention included 6, 9, 19 and 21. One of the limitations of the FATCOD tool was the use of double negatives in its wording which may offer an explanation for these four items. Or alternatively, as reported by Twigg and Lynn, the end of life simulation may induce anxiety in those students who have no previous experience with death [25]. However without information on the students’ previous experience this cannot be confirmed. Two students also had a decrease in their FATCOD scores post the simulated intervention (Table 2). The rationale for the decrease in mean item scores could also apply to these two cases; however it is worth noting that both participants still had relatively high post intervention scores indicating a positive attitude towards end of life care.

There are limitations to this study which are important to acknowledge. This was a small pilot study undertaken in one HEI therefore caution must be taken when applying statistical inference. The pre and post FATCOD questionnaires were anonymous which meant it was not possible to look at differences in attitudes between the two professions and socio-demographic data was not collected to determine the influence of previous death experience and/or education. Socio-demographic variables have been examined by previous authors investigating attitudes towards end of life care using the FATCOD tool [13, 17, 26]. Their results revealed that age, gender and prior experience of death can impact on the attitudes of medical students [17], nursing students [13] and registered nurses’ [26] towards end of life care. These would certainly warrant further study.

As mention previously, the FATCOD items contained double negatives which may have introduced a source of error. Additionally, there was no control group to compare the end of life simulation against standard teaching. There may also have been potential for participant bias due to the voluntary nature of the study, as those students who participated may have had a keen interest in end of life care or simulation based learning. This was only a pilot study therefore replication with larger numbers is necessary to confirm its findings. Further research is warranted into the use of end of life simulation with medical and nursing students and from an IPE perspective, including qualitative studies with both professions to gain an insight into the student experience. Ideally, the students who participated in this study could be followed up to determine if the impact of the end of life simulation translated into improved confidence and competence in the practice setting. This would certainly warrant further study.

## Conclusion

End of life care needs to be delivered by healthcare professionals with the skills, knowledge and experience to meet the needs of patients and their families adequately [27]. The results of this study support the growing body of literature which suggests that active, experiential learning in the form of simulation helps improve attitudes of undergraduate nursing and medical students towards end of life care. In the absence of experience in clinical placement of end of life care, exposure to experiential learning is vital not only to prepare nursing students and medical students for their professional role, but to ensure that high quality end of life care is afforded to all patients and their families.
